# Updated Genome Sequence of the Facultative Methanotroph *Methylocystis* sp. Strain SB2

**DOI:** 10.1128/mra.00188-22

**Published:** 2022-05-16

**Authors:** Christina S. Kang-Yun, Jin Chang, Jeremy D. Semrau

**Affiliations:** a Department of Civil and Environmental Engineering, University of Michigan, Ann Arbor, Michigan, USA; University of Southern California

## Abstract

The genome of *Methylocystis* sp. strain SB2, which was previously isolated from a spring bog in southeast Michigan, was sequenced using long-read sequencing technology. This new sequencing and assembly effort yielded a complete assembly of the genome in a single contig.

## ANNOUNCEMENT

*Methylocystis* sp. strain SB2 was isolated from a spring bog near Ann Arbor, Michigan, in 2006 (1). The genome of this facultative methanotroph was first reported in 2013, but the initial assembly resulted in 158 contigs (1). To generate a better assembly, *Methylocystis* sp. strain SB2, which was both stored as frozen stocks at −80°C and maintained on nitrate mineral salt (NMS) agar plates (2), was grown again in liquid NMS medium at 30°C as described earlier (1). DNA was extracted using Genomic-tip 500/G (Qiagen, Hilden, Germany). Libraries were prepared using ligation sequencing and native barcoding expansion kits (SQK-LSK109 and EXP-NBD104, respectively; Oxford Nanopore Technologies, Littlemore, UK), and size selection was not done prior to sequencing. All kits were used following the manufacturers’ protocols. Sequencing was performed on the GridION X5 platform using a MinION flow cell (FLO-MIN106; Oxford Nanopore Technologies) at the University of Michigan Advanced Genomics Core and produced 196,551 reads that passed quality control. The minimum, maximum, and mean lengths of these reads were 96 bp, 156,444 bp, and 7,701 bp, respectively, with an *N*_50_ value of 14,594 bp. Base calling was performed using Guppy (v4.2.3) (3). Sequence quality was assessed using FastQC (v0.11.9) (4) before and after trimming. The reads were trimmed using Porechop (v0.2.4) (5) and then were filtered using Filtlong (v0.2.0) (6); 133,502 reads remained after filtering, with minimum, maximum, and mean lengths of 1,710 bp, 156,375 bp, and 10,637 bp, respectively, and an *N*_50_ value of 15,552 bp. The reads were then used to assemble the genome using Trycycler (v0.4.1) (7). Briefly, multiple assemblies were constructed using Flye (v2.9-b1768) (8), Miniasm (v0.3-r179) (9) with Minipolish (v0.1.2) (10), and Raven (v1.6.1) (11) and were collectively processed to produce a consensus assembly. The consensus assembly was then polished using Medaka (v1.0.3) (12). The final contig was annotated using the National Center for Biotechnology Information (NCBI) Prokaryotic Genome Annotation Pipeline (PGAP) (v5.3) (13). Default parameters were used for all software unless otherwise specified.

The complete genome of *Methylocystis* sp. strain SB2 was found to consist of one chromosome of 3.54 Mbp, with general features as described in Table 1. The average nucleotide identity (ANI), as determined using the OrthoANIu algorithm (v20210707) (14), between the updated and original genomes of *Methylocystis* sp. strain SB2 was 99.97% (1). The digital DNA-DNA hybridization (dDDH) value between these genomes, as determined using the Genome BLAST Distance Phylogeny algorithm (v3.0) (15), was 99.8%.

**TABLE 1:** General features of *Methylocystis* sp. strain SB2 genome

Parameter	Finding for strain SB2
Complete genome size (bp)	3,543,002
No. of contigs	1
G+C content (%)	62.7
Total no. of coding sequences	3,292
No. of rRNA genes (16S, 23S, and 5S)	3
No. of tRNA genes	48
No. of GridION reads	196,551
SRA accession no.	SRR17701709
GenBank accession no. for annotated genome sequence	CP091318.1
ANI with original genome^*a*^ (%)	99.97
dDDH with original genome^*a*^ (%)	99.80

a GenBank accession number AYNA00000000.1.

### Data availability.

The annotated genome sequence has been deposited in GenBank under accession number CP091318.1. The raw reads have been deposited in the NCBI Sequence Read Archive (SRA) under accession number SRR17701709.
